# Supplementary material for: Attenuated Mycobacterium tuberculosis vaccine protection in a low-dose murine challenge model
Source: iScience. 2023 May 28;26(6):106963. doi: 10.1016/j.isci.2023.106963 (PMC10291467; doi:10.1016/j.isci.2023.106963)
Supplement: Document S1. Figures S1–S3 [file mmc1.pdf]

iScience, Volume *26*

Supplemental information

Attenuated *Mycobacterium tuberculosis* vaccine protection in a low-dose murine challenge model

Samuel J. Vidal, Daniel Sellers, Jingyou Yu, Shoko Wakabayashi, Jaimie Sixsmith, Malika Aid, Julia Barrett, Sage F. Stevens, Xiaowen Liu, Wenjun Li, Courtney R. Plumlee, Kevin B. Urdahl, Amanda J. Martinot, and Dan H. Barouch

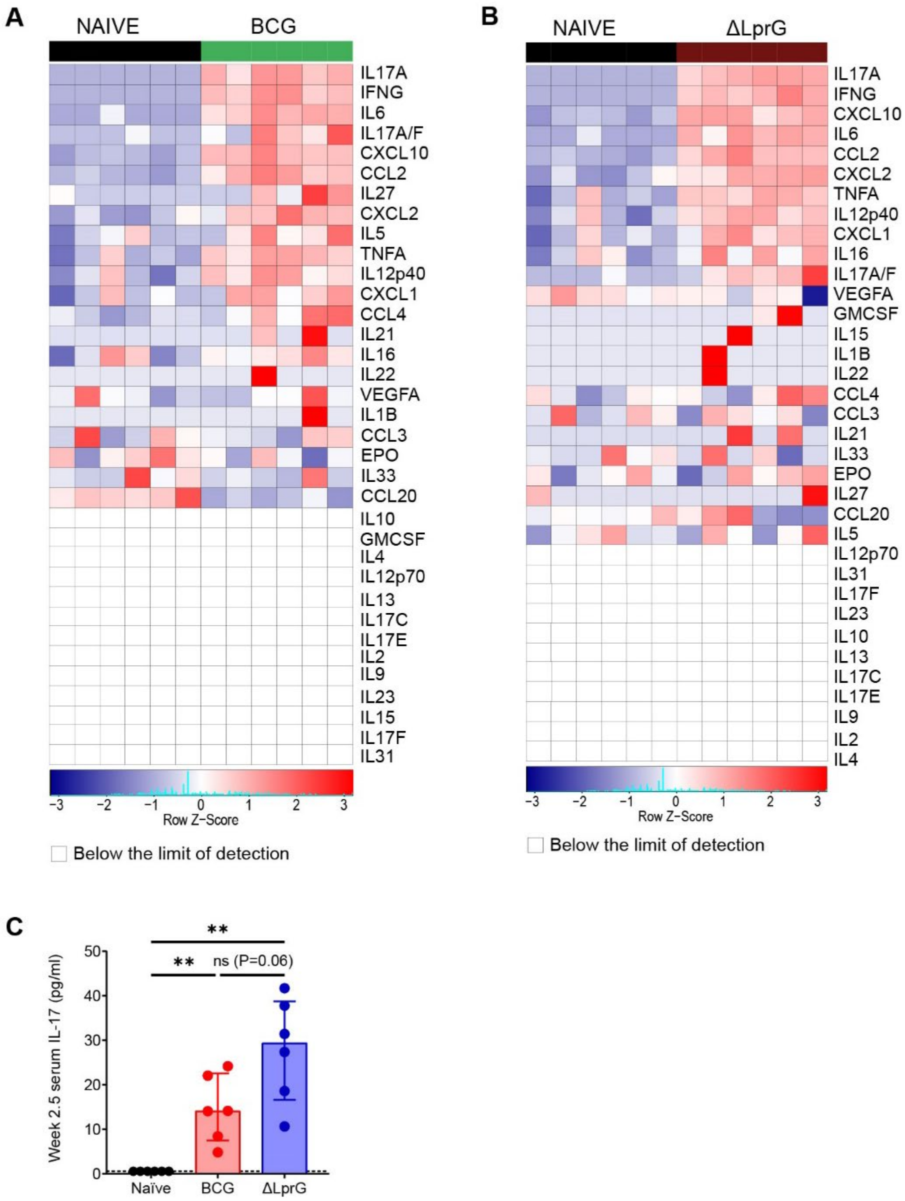

Figure S1. Induction of serum cytokines among naïve and BCG- and ΔLprG-vaccinated C3HeB/FeJ mice, related to Figure 1. A cohort of C3HeB/FeJ mice (n=18) was divided into naïve (n=6), BCG (n=6), and ΔLprG (n=6) groups. Vaccines were administered at week 0 and serum cytokines were measured at week 2.5. Heat map comparing serum cytokine levels between the naïve and BCG groups (A). Heat map comparing serum cytokine levels between the naïve and ΔLprG groups (B). Serum IL-17A levels among the three groups (C). P values represent pair-wise Mann-Whitney U tests. ** represents P<0.01.

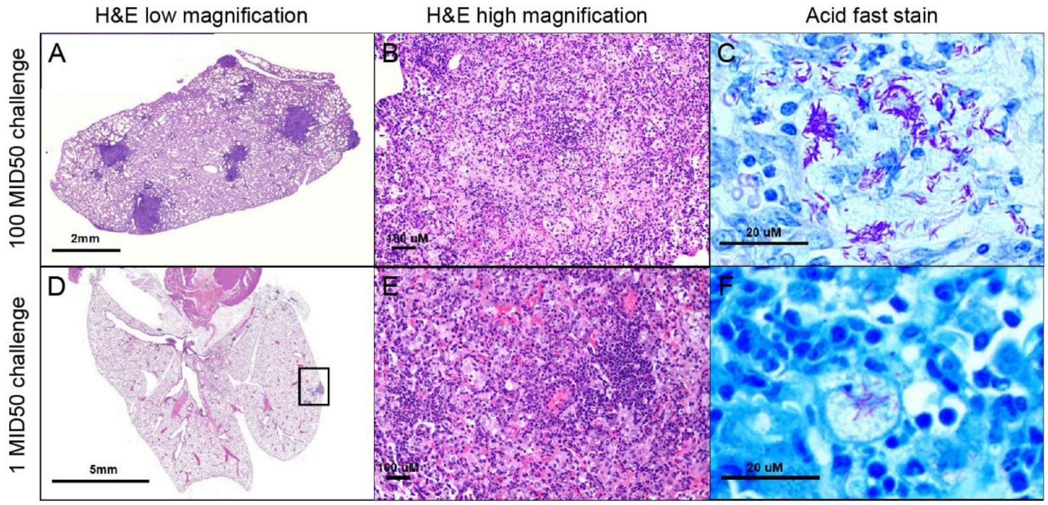

Figure S2. Histopathology of lung tissues from C3HeB/FeJ mice following 1 MID50 or 100 MID50 H37Rv challenge, related to Figure 2. Representative low-magnification H&E stain (A), high-magnification stain (B), and acid fast stain (C) of lung tissues from C3HeB/FeJ mice 4 weeks after 100 MID50 H37Rv challenge. Representative low-magnification H&E stain (D), high-magnification stain (E), and acid fast stain (F) of lung tissues from C3HeB/FeJ mice 4 weeks after 1 MID50 H37Rv challenge.

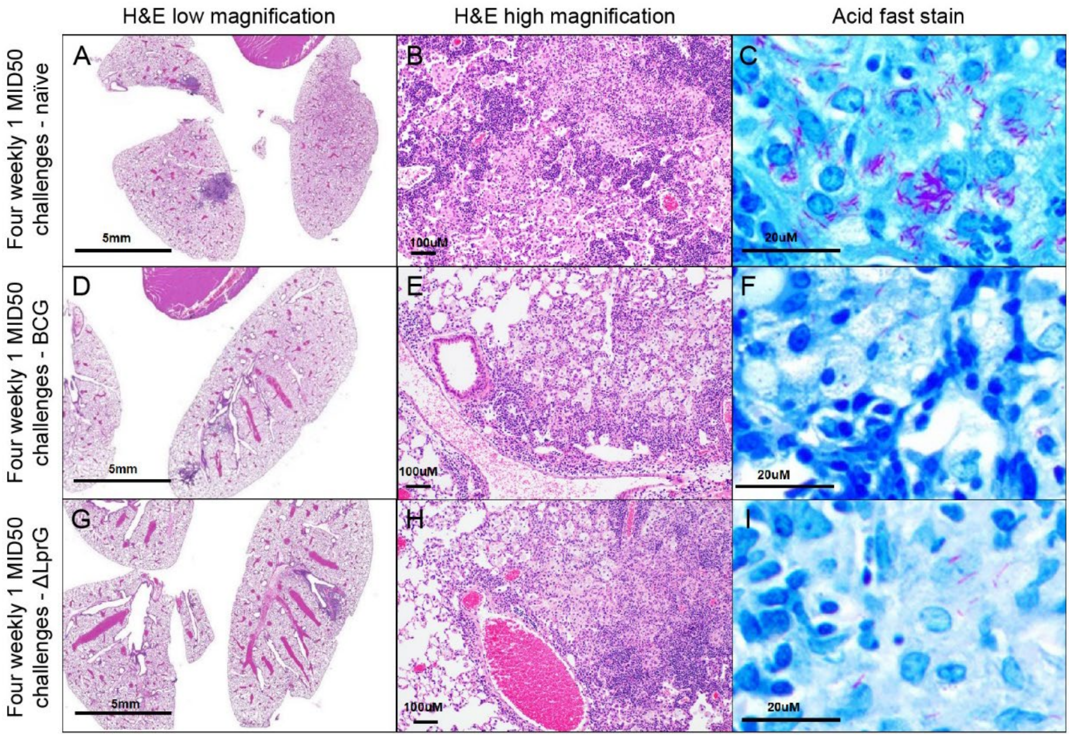

Figure S3. Histopathology of lung tissues from naïve and BCG- and ΔLprG-vaccinated C3HeB/FeJ following four weekly 1 MID50 H37Rv challenges, related to Figure 4. Representative low-magnification H&E stains (A, D, and G), high-magnification stains (B, E, and H), and acid fast stains (C, F, and I) of lung tissues from C3HeB/FeJ mice 4 weeks after 4 weekly 1 MID50 H37Rv challenges under naïve (A-C), BCG- (D-F), and ΔLprG-vaccinated (G-I) conditions.
